# Correlation between related inflammation indicators and the degree of liver fibrosis in patients with chronic hepatitis B

**DOI:** 10.1097/MD.0000000000043545

**Published:** 2025-08-01

**Authors:** Jiayan Shen, Shuncong Dai, Yingshuang Huang, Xiuqin Fan, Shuhui Zhao, Zejun Wang

**Affiliations:** a Department of Gastroenterology, Linping Hospital of Integrated Traditional Chinese and Western Medicine, Hangzhou, Zhejiang Province, PR China; b Department of Ultrasound, Linping Hospital of Integrated Traditional Chinese and Western Medicine, Hangzhou, Zhejiang Province, PR China.

**Keywords:** chronic hepatitis B (CHB), liver fibrosis, neutrophil-to-lymphocyte ratio (NLR), platelet-to-lymphocyte ratio (PLR), systemic immune inflammation index (SII)

## Abstract

This study aims to investigate the values of related inflammation indicators in predicting the degree of liver fibrosis in patients with chronic hepatitis B (CHB). A 1-year retrospective study was conducted. The 120 CHB patients in the case group were divided into the non-fibrosis group (n = 26), mild-moderate fibrosis group (n = 34), severe fibrosis group (n = 27), and cirrhosis group (n = 33) based on the results of liver transient elastography. A total of 28 healthy individuals were included in the control group. Systemic immune inflammation index (SII), platelet-to-lymphocyte ratio (PLR), neutrophil-to-lymphocyte ratio (NLR), lymphocyte-to-monocyte ratio, and monocyte-to-lymphocyte ratio were observed and compared. There was a significant correlation between liver fibrosis and SII, NLR, and PLR (*P* < .001), with the strongest correlation between liver fibrosis and SII (*r* = −0.952). With an area under the receiver operating characteristic curve of 0.982, an SII ≤ 353.605 had the best sensitivity accuracy (97.9%) for the detection of any grade of liver fibrosis. The 5 combined indicators had the highest specificity accuracy (96.2%). In limited-resource settings, the SII could serve as a new strategy for predicting the degree of liver fibrosis in CHB patients and was superior to the PLR and NLR.

## 1. Introduction

Hepatitis B virus (HBV) infection is a global epidemic and chronic hepatitis B (CHB) has become a major public health problem worldwide. According to the World Health Organization, an estimated 254 million people live with hepatitis B by 2022, with 1.2 million new infections each year.^[1]^ In particular, HBV infection leads to CHB in hundreds of millions of people and is the most common cause of liver cirrhosis, liver cancer, and hepatitis-related deaths. Notably, chronic viral hepatitis can lead to hepatocellular carcinoma (HCC), which accounts for 80% of all liver cancer cases and is the third most common cause of cancer-related deaths worldwide.^[2,3]^ Liver fibrosis, which is associated with significant morbidity and mortality, is necessary for CHB to develop into cirrhosis and is an important contributor to the global disease burden.^[4]^ Liver fibrosis is the most common pathological outcome of various chronic liver diseases, and it epitomizes the universal response of the liver to acute or chronic liver injury. After acute liver damage, parenchymal cells replace necrotic and apoptotic cells by the process of regeneration; however, during chronic liver injury, the regenerative response gradually fails, and the extracellular matrix leading to liver fibrosis increasingly substitutes for hepatocytes.^[5]^ Early use of effective anti-hepatic fibrosis therapy can control and delay disease progression, reduce mortality, and improve patient prognosis.^[6]^ At present, liver biopsy is still the gold standard for the diagnosis of liver fibrosis in clinical practice. However, as an invasive diagnostic method, it is difficult to use widely and cannot be used as a continuous monitoring method because of its poor tolerance, high operational difficulty, and many complications.^[7]^

The systemic immune inflammation index (SII) and blood count-derived inflammatory markers, such as the platelet-to-lymphocyte ratio (PLR), neutrophil-to-lymphocyte ratio (NLR), lymphocyte-to-monocyte ratio (LMR), and monocyte-to-lymphocyte ratio (MLR), have gained increasing interest in recent years. PLR is a marker of inflammation which established in liver fibrosis, Covid-19 infection, and metastatic colorectal cancer.^[8–10]^ Similarly, the inflammatory role of NLR has been reported in gastric cancer, acute anterior uveitis, and hepatitis C virus (HCV)-related liver disease.^[11,12]^ Furthermore, in a younger population undergoing humeral shaft fracture osteosynthesis, postoperative NLR as inflammatory markers is strongly correlated with the degree of surgical trauma experienced.^[13]^ MLR and LMR have been noted in predicting mortality and prognosis of malignancy.^[14–16]^ SII is a new relevant inflammatory index based on routine blood results and has been suggested as a marker of inflammatory burden in diabetic nephropathy, and solid tumors.^[17–19]^ They have been proven to be associated with local immune responses and systemic inflammation in the human body.^[20,21]^ Flaviu Moldovan have shown that the SII and NLR may increase the accuracy of diagnosing periprosthetic joint infections when used in conjunction with other well-established parameters.^[22]^ Meanwhile, inflammation is generally believed to be the core mechanism underlying liver fibrosis progression.^[23]^ Recent studies on the mechanisms of fibrosis have focused on hepatic stellate cells (HSC), which become fibrogenic myofibroblasts during injury through “activation.”^[24]^ Routine blood examination can comprehensively reflect the inflammatory state of the body. Recent studies have suggested that the PLR, NLR, LMR, MLR, and other relevant inflammatory indicators can reflect the progression of CHB and the pathological changes in liver cirrhosis.^[21,25]^ However, there are still differences in these research results, and there is a lack of applied research on SII in the evaluation of liver fibrosis. Therefore, screening for liver fibrosis is essential because it identifies patients at risk and guides treatment decisions to avoid the progression of CHB to liver cirrhosis and even hepatic carcinoma.

Based on the current situation, the values of the SII and the abovementioned related inflammatory indicators (PLR, NLR, LMR, and MLR) in predicting the degree of liver fibrosis in CHB patients were studied and analyzed in this study.

## 2. Materials and methods

### 2.1. Study population

A retrospective study was conducted on patients admitted for CHB at Linping Hospital of Integrated Traditional Chinese and Western Medicine between January 2023 and December 2023. The inclusion criteria were patients older than 18 years of age who were diagnosed with CHB in the aforementioned timeframe and for whom data regarding general information and laboratory investigations were available. All patients met the diagnostic criteria in the Guidelines for the Prevention and Treatment of Chronic Hepatitis B (2022 updated version) formulated by the Chinese Society of Hepatology and the Chinese Society of Infectious Diseases. The following patients were excluded: patients with malignant tumors, cardiovascular or cerebrovascular accidents, endocrine system diseases, autoimmune diseases, blood system diseases, or other acute or chronic systemic infections other than HBV infection; patients with severe trauma, surgery, or transfusion and transfusion of blood components within 1 month before enrollment; patients with liver fibrosis not caused by HBV infection; and pregnant or lactating patients. The study eventually included 148 participants who were divided into 2 groups: 120 patients with CHB as the case group and 28 healthy individuals who underwent physical examination as the control group. In the case group, 26 patients were included in the non-fibrosis group, 34 patients were included in the mild-moderate fibrosis group, 27 patients were included in the severe fibrosis group, and 33 patients were included in the cirrhosis group based on the results of transient elastic hardness examination. This study was approved by the Ethics Committee of the Linping Hospital of Integrated Traditional Chinese and Western Medicine, and all participating patients signed informed consent forms.

### 2.2. Data collection

Data were collected from the hospital’s electronic databases. Information regarding demographics (age and sex), and laboratory parameters was extracted for each patient. Fasting peripheral venous blood samples from both groups were collected in the morning. The sampling time of the case group was the day after admission, and that of the control group was the day of the physical examination. The samples were sent to the laboratory for routine blood and liver function tests within 2 hours of blood collection. The absolute platelet count, absolute lymphocyte count, absolute neutrophil count, and absolute monocyte count were measured by complete blood count using the hematology analyzer MINDRAY BC-7500 and were presented as ×10^9^/L, and alanine aminotransferase (ALT) and aspartate aminotransferase (AST) were detected using a Beckman Coulter AU 5800.

### 2.3. Biomarkers

Five ratios were calculated based on blood count-derived inflammatory markers and were considered prognostic factors:

1. SII = ANC × APC/ALyC, where ANC = absolute neutrophil count, APC = absolute platelet count, ALyC = absolute lymphocyte count;
2. PLR = platelet-to-lymphocyte ratio, calculated by dividing platelet count by lymphocyte count;
3. NLR = neutrophil-to-lymphocyte ratio, calculated by dividing neutrophil count by lymphocyte count;
4. LMR = lymphocyte-to-monocyte ratio, calculated by dividing lymphocyte count by monocyte count;
5. MLR = monocyte-to-lymphocyte ratio, calculated by dividing monocyte count by lymphocyte count.

### 2.4. Study outcome

The primary aim of our study was to assess whether blood count-derived inflammatory markers could serve as predictors of liver fibrosis severity in patients with CHB. The degree of liver fibrosis was estimated using transient elastography of the liver. Patients in the case group underwent transient elastic hardness examination, stage F0F1 was observed when the liver hardness was <7.3 kPa, stage F2 was observed when the liver hardness was <9.7 kPa, stage F2F3 was observed when the liver hardness was <12.4 kPa, stage F3F4 was observed when the liver hardness was 12.4 to 17.5 kPa, and stage F4 was observed when the liver hardness was ≥17.5 kPa. Patients with F0F1 stage were classified into the non-fibrosis group, patients with F2 stage and F2F3 stages were classified into the mild-moderate fibrosis group, patients with F3F4 stage were classified into the severe fibrosis group, and patients with F4 stage were classified into the cirrhosis group. We investigated whether noninvasive fibrosis markers, such as SII, PLR, NLR, LMR, and MLR, were efficient in predicting liver fibrosis in patients with CHB.

### 2.5. Statistical analysis

Statistical analysis was performed using SPSS version 25.0 (IBM, Armonk) and R version 4.3.2 (R Foundation for Statistical Computing, Vienna, Austria). Normality was tested using the Shapiro–Wilk test. Continuous variables were expressed as the median with interquartile range or mean with standard deviation, whereas for categorical variables, the absolute count (n) and proportions were given. Categorical variables were compared using the chi-square test, while analysis of variance was used for continuous variables if they were normally distributed; otherwise the Kruskal–Wallis (K–W) test was used. The Student–Newman–Keuls test was used for pairwise comparison. Correlations were evaluated using Spearman’s correlation coefficient. The performance of inflammatory indicators in predicting liver fibrosis severity was assessed using receiver operating characteristic (ROC) curve analysis and the area under the ROC curve (AUC). The optimal cutoff values for relevant systemic inflammatory markers were determined from the ROC curve using the Youden index. *P* < .05 was considered statistically significant throughout all the analyses.

## 3. Results

In this study, 148 adults were included based on the inclusion and exclusion criteria: 62.16% were men, and 37.84% were women. As depicted in Table 1, there were no significant differences in sex, age, ALT, or AST among the 5 groups. The SII showed significant differences in pairwise comparisons among the 5 groups (*P* < .01), and there was a distinct trend toward a higher SII with a decreasing degree of liver fibrosis, as shown in Table 1. The cirrhosis group had the lowest SII, whereas the control group had the highest. Compared with that in the liver fibrosis group, regardless of the degree of fibrosis, the PLR tended to increase in the non-fibrosis and control groups (*P* < .01), but there was no significant difference between the mild-moderate fibrosis group and the severe fibrosis group. Similarly, the cirrhosis group had a significantly lower PLR than the other groups (*P* < .01). The NLR in the non-fibrosis group was only higher than that in the severe fibrosis and cirrhosis groups (*P* < .01), and the NLR in the mild-moderate group was only higher than that in the cirrhosis group (*P* < .01). However, the NLR in the case group was statistically lower than that in the control group (*P* < .01). The LMR in the cirrhosis group was higher than that in the non-fibrosis, mild-moderate fibrosis, and control groups (*P* < .01). The MLR in the severe fibrosis group was higher than that in the cirrhosis group and was lower than that in the control group.

**Table 1:** Comparison of the clinical characteristics and related inflammation indicators among the 5 groups.

Variables	Non-fibrosis (n = 26)	Mild/moderate fibrosis (n = 34)	Severe fibrosis (n = 27)	Cirrhosis (n = 33)	Controls (n = 28)	F/H/X^2^	*P*
Gender							
F	13 (50%)	9 (26.47%)	11 (40.74%)	13 (39.39%)	10 (35.71%)	3.687	.45
M	13 (50%)	25 (73.53%)	16 (59.26%)	20 (60.61%)	18 (64.29%)		
Age (yr)	40.69 ± 8.44^a^	45.06 ± 10.02^a^	45.19 ± 9.48^a^	42.79 ± 10.35^a^	42.32 ± 8.43^a^	1.157	.333
ALT (U/L)	21.00 (15.90–31.25)^a^	28.50 (21.50–38.75)^a^	22.00 (15.00–31.00)^a^	28.00 (18.50–40.50)^a^	27.50 (17.00–32.32)^a^	5.944	.203
AST (U/L)	21.20 (18.00–26.25)^a^	23.00 (20.00–32.25)^a^	23.00 (19.00–26.00)^a^	24.00 (21.00–29.50)^a^	23.00 (19.25–26.75)^a^	5.387	.250
SII	425.39 ± 69^d^	320.78 ± 23.42^c^	254.97 ± 17.28^b^	184.62 ± 38.98^a^	660.14 ± 120.37^e^	244.467	<.001
PLR	128.45 ± 31.67^a^	107.46 ± 17.83^b^	98.97 ± 25.42^bc^	76.27 ± 23.86^d^	151.95 ± 27.26^e^	39.046	<.001
NLR	2.00 (1.78–2.29)^abe^	1.72 (1.54–2.07)^bc^	1.50 (1.19–1.67)^cd^	1.21 (1.02–1.53)^d^	2.69 (2.32–3.51)^e^	89.583	<.001
LMR	4.72 ± 1.67^ae^	4.84 ± 1.46^abe^	5.44 ± 1.71^ac^	6.13 ± 2.97^cd^	3.70 ± 1.22^e^	6.478	<.001
MLR	0.21 (0.18–0.28)^abc^	0.20 (0.18–0.25)^abc^	0.19 (0.16–0.23)^a^	0.18 (0.13–0.26)^bc^	0.29 (0.22–0.35)^c^	22.066	<.001

The same alphabet in the footnote means nonsignificant between 2 groups, otherwise significant.

ALT = alanine aminotransferase, AST = aspartate aminotransferase, F = female, LMR = lymphocyte-to-monocyte ratio, M = male, MLR = monocyte-to-lymphocyte ratio, NLR = neutrophil-to-lymphocyte ratio, PLR = platelet-to-lymphocyte ratio, SII = systemic immune inflammation index.

The correlations between related inflammatory indicators and the degree of liver fibrosis are shown in Table 2. After excluding the data of the control group, Spearman correlation analysis revealed that the degree of liver fibrosis in CHB patients was negatively correlated with the SII, PLR, and NLR (*P* < .01), and was also negatively correlated with the MLR (*P* < .05), but positively correlated with LMR (*P* < .05).

**Table 2:** Correlation between the related inflammatory indicators and the degree of liver fibrosis.

Variables	Different cirrhosis groups (Ordinal)	
	Correlation coefficient	*P*
SII	−0.952**	<.001
PLR	−0.627**	<.001
NLR	−0.650**	<.001
LMR	0.186*	.042
MLR	−0.187*	.041

Analyzed by Spearman’s correlation.

LMR = lymphocyte-to-monocyte ratio, MLR = monocyte-to-lymphocyte ratio, NLR = neutrophil-to-lymphocyte ratio, PLR = platelet-to-lymphocyte ratio, SII = systemic immune inflammation index.

* *P* < .05.

** *P* < .01.

**Figure 1.**
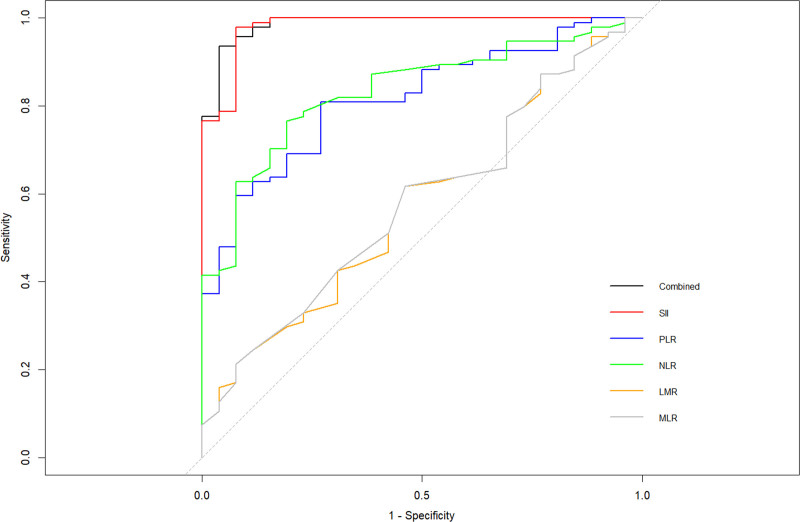
ROC curves of the related inflammation indicators in prediction of the liver fibrosis. LMR = lymphocyte-to-monocyte ratio, MLR = monocyte-to-lymphocyte ratio, NLR = neutrophil-to-lymphocyte ratio, PLR = platelet-to-lymphocyte ratio, ROC = receiver operating characteristic, SII = systemic immune inflammation index.

The values of the related inflammation indicators for the prediction of liver fibrosis are presented in Figure 1 and Table 3. ROC curves were used to analyze the value of inflammatory indicators in predicting liver fibrosis, with the mild-moderate fibrosis, severe fibrosis and cirrhosis groups as the case group, and the non-fibrosis group as the control group. The results showed that only the LMR and MLR had no predictive significance (*P* > .05), whereas the AUCs of the SII, PLR, and NLR in predicting liver fibrosis were statistically significant (*P* < .01), with AUCs of 0.982, 0.816, and 0.834, respectively. Moreover, compared with PLR and NLR, SII showed better predictive sensitivity and specificity, which were 97.9% and 92.3%, respectively, with a cutoff at 353.605. When the 5 indicators were combined, the AUC was 0.986, with the sensitivity and specificity of 93.6% and 96.2%, respectively.

**Table 3:** The values of the related inflammatory indicators in prediction of the severe fibrosis and the liver cirrhosis.

Variables	AUC	SE	*P*	95% CI	Cutoff value	Sensitivity (%)	Specificity (%)	PPV (%)	NPV (%)
Combined	0.986	0.009	<.001	0.9679–1.0000	0.810	93.6	96.2	98.9	80.6
SII	0.982	0.012	<.001	0.9572–1.0000	353.605	97.9	92.3	97.9	92.3
PLR	0.816	0.042	<.001	0.7343–0.8975	111.590	80.9	73.1	91.6	51.4
NLR	0.834	0.040	<.001	0.7561–0.9121	1.750	76.6	80.8	93.6	50.0
LMR	0.574	0.062	.229	0.4532–0.6958	4.875	61.7	53.8	82.9	28.0
MLR	0.580	0.061	.192	0.4598–0.7002	0.205	61.7	53.8	82.9	28.0

AUC = area under curve, CI = confidence interval, LMR = lymphocyte-to-monocyte ratio, MLR = monocyte-to-lymphocyte ratio, NLR = neutrophil-to-lymphocyte ratio, NPV = negative predictive value, PLR = platelet-to-lymphocyte ratio, PPV = positive predictive value, SE = standard error, SII = systemic immune inflammation index.

## 4. Discussion

The present study indicates that the related inflammation indicator SII can be a predictor of the degree of liver fibrosis in patients with CHB. The PLR and NLR also have a certain predictive value; however, the value of LMR and MLR in predicting the degree of liver fibrosis is not well expected. When these 5 indicators are combined, the prediction effect can be improved to a certain extent.

Our study revealed that the higher the degree of liver fibrosis, the lower the SII, PLR, and NLR, indicating that these 3 indicators were negatively correlated with the degree of liver fibrosis. Various noninvasive tests have been validated to assess liver fibrosis severity, while blood count-derived inflammatory markers have been proven to be reliable in reflecting the inflammatory status of patients with CHB.^[26]^ The SII, reported by Hu et al in 2014,^[27]^ is a novel marker of inflammation, and liver fibrosis is also associated with inflammation. The value of SII in predicting the prognosis of patients with liver cancer has been verified in recent years, especially in patients who have undergone hepatectomy.^[19,28]^ The SII has also been applied to predict and evaluate the prognosis of patients with gastric cancer, esophageal cancer, non-small cell lung cancer, cardiovascular disease, etc.^[18,29–31]^ However, the relationship between the SII and hepatic fibrosis remains unclear. Our study showed that the SII was significantly negatively correlated with the degree of liver fibrosis in CHB patients. However, Xie et al^[32]^ reported that the SII was positively correlated with hepatic steatosis, but there was no significant correlation between SII levels and liver fibrosis, which was derived from the National Health and Nutrition Examination Survey dataset nationally representing adults in the United States. The difference is that our study predicted the degree of liver fibrosis after chronic HBV infection, whereas their study predicted liver fibrosis after steatosis.

Although our study revealed that PLR and NLR have certain predictive value for the degree of liver fibrosis in patients with CHB, studies with larger sample sizes are still needed for further verification. The indicative role of PLR and NLR in solid tumors such as liver cancer, gastric cancer, and colorectal cancer, has been well validated in past researches.^[10,11,33]^ In addition, PLR and NLR have prognostic significance in nonneoplastic diseases such as infectious diseases, idiopathic acute anterior uveitis, appendicitis, and thrombosis-related diseases.^[12,31,34]^ The relationships between PLR, NLR and liver fibrosis have been explored in other studies, with conflicting results. Alsebaey et al^[35]^ reported that PLR is useful in distinguishing HCV positive patients with significant fibrosis, unlike NLR, which was comparable; however, in a study published by Kara et al,^[36]^ NLR was not associated with the severity of liver fibrosis. Moreover, Meng et al^[37]^ reported that PLR was related to the severity of HCV-related liver disease and could predict the viral clearance effect of chronic hepatitis C after interferon ribavirin treatment. However, in regard to HBV infection, Wang et al^[38]^ reported that the severity of liver fibrosis affected the predictive value of the PLR and NLR in patients with HBV-related HCC, and the study showed that the PLR and NLR only had good predictive value in HBV-HCC patients without severe fibrosis. In contrast, our study revealed no significant difference in the PLR between the mild-moderate and the severe fibrosis groups; however, the PLR has a certain diagnostic value for distinguishing the presence or absence of liver fibrosis. The NLR has certain diagnostic value for the evaluation of liver cirrhosis. Despite the significant role of inflammatory markers, such as PLR and NLR, in infectious diseases, the underlying mechanism remains unknown. In addition, the body is prone to changes in lymphocytes, neutrophils, platelets and other hematological indicators during viral infection, and such changes are affected by many factors, which indirectly affect the PLR and NLR.

In our study, although MLR and LMR may be pathophysiologically and clinically relevant in patients with CHB, their predictive power was limited. No significant differences in AST or ALT levels were noted among the 5 study groups. Since ALT and AST can be easily influenced by various factors, such as diet, living habits, and metabolic status,^[39]^ these 2 indices are not individually used to assess liver fibrosis in general. LMR and MLR have been found to have clinical significance in pancreatic cancer, esophageal squamous cell carcinoma, urothelial carcinoma of the bladder, etc.^[14–16]^ Tiucă et al^[40]^ also revealed that patients with psoriasis vulgaris from high risk of significant fibrosis group had higher values of the MLR than low risk of significant fibrosis group (*P* < .001), which further significantly correlated with fibrosis severity (*P* < .001).

Furthermore, this study showed that the SII has a better correlation with liver fibrosis and can be used to predict the degree of liver fibrosis, compared with PLR, NLR, MLR, and LMR. In addition, the SII, PLR, and NLR were all good indicators of liver fibrosis, with an AUC > 0.60, and the SII proved to be of particular help in predicting the degree of liver fibrosis in patients with CHB, with an AUC of 0.982, and was superior to PLR and NLR. Although the calculation of SII is still based on the results of routine blood examination, it comprehensively considers the synergistic change trend of neutrophils and platelets relative to that of lymphocytes. According to a previous study, SII is a novel marker of inflammation, and in the case of CHB infection, the host immune response induces extensive hepatocyte damage, leading to fibrosis.^[41]^ During fibrosis, crosstalk between parenchymal and non-parenchymal cells, activation of different immune cells and signaling pathways, and the release of several inflammatory mediators take place, resulting in inflammation.^[5]^ Previous studies have confirmed that inflammatory cells and factors such as neutrophils, macrophages, mast cells, and transforming growth factor β, platelet-derived growth factor, and tumor necrosis factor α could promote the formation and development of liver fibrosis by regulating physiological and pathological states.^[42]^ Excessive inflammation drives HSC activation and HSCs are the most important contributors to liver fibrosis. Persistent viral exposure activates different immune cells, producing numerous pro-inflammatory cytokines and fibrogenic mediators, and recruiting other immune cells to the liver driving hepatic inflammation.^[5]^ Our results demonstrated a significant negative relationship between the SII and the degree of liver fibrosis; in other words, the SII could serve as a new indicator for predicting the degree of liver fibrosis in CHB patients to facilitate the early identification of liver fibrosis.

Our study is not without its limitations. The main limitation of this study lies in its single-institution retrospective nature, and whether the Linping Hospital of Integrated Traditional Chinese and Western Medicine was a suitable source of data was unknown, due to its regional and limited patient source. Furthermore, the relatively small sample size limited the generalizability of our findings. In addition, the use of transient elastography rather than liver biopsy placed certain restrictions on this study, although several studies have demonstrated the extremely high accuracy of transient elastography. Despite these limitations, related inflammation indicators have attracted increased interest taking into account the noninvasiveness, easy accessibility, and low cost of these ratios. Our study clarified the predictive value of related inflammation indicators for the degree of liver fibrosis in patients with CHB. The exploration of the mechanism of inflammatory indicators and HBV infection, and the establishment of normal boundaries of related inflammation indicators will provide better insights for predicting the degree of liver fibrosis.

## 5. Conclusion

The SII, PLR, and NLR could serve as new strategies for predicting the degree of liver fibrosis in CHB patients, and the SII proved to be of particular help in predicting significant liver fibrosis. Additional large-scale prospective investigations are required to confirm these findings.

## Author contributions

**Conceptualization:** Jiayan Shen, Zejun Wang.

**Data curation:** Jiayan Shen, Shuncong Dai, Yingshuang Huang, Xiuqin Fan, Shuhui Zhao.

**Formal analysis:** Jiayan Shen, Shuncong Dai, Shuhui Zhao, Zejun Wang.

**Investigation:** Jiayan Shen, Zejun Wang.

**Methodology:** Jiayan Shen, Zejun Wang.

**Supervision:** Zejun Wang.

**Writing – original draft:** Jiayan Shen, Shuncong Dai, Yingshuang Huang, Xiuqin Fan.

**Writing – review & editing:** Jiayan Shen, Zejun Wang.
